# 394. Descriptive Evaluation of Epidemiology and Microbiology of Patients with COVID-19 Pre/post Implementation of Corticosteroids as Standard of Care

**DOI:** 10.1093/ofid/ofab466.595

**Published:** 2021-12-04

**Authors:** Goran Flajc, Ahmed Zaki

**Affiliations:** Baylor St. Luke’s Medical Center, Houston, Texas

## Abstract

**Background:**

The coronavirus disease 2019 (COVID-19) pandemic continues to present a significant global public health concern. As of June 2021, nearly 174 million cases of SARS-CoV-2 infection worldwide have been reported to the World Health Organization. Rigorous data on the efficacy of corticosteroids have now established its role as standard of care (SOC). Less recognition has been given to corticosteroid therapy and its association with risk of infection especially in those who are critically ill and prone to nosocomial pathogens.

**Methods:**

This is a retrospective study of mechanically ventilated patients with COVID - 19 from March 2020 to September 2020 at a single center. The primary endpoint for this study was description of microbiology and epidemiology of secondary infections and co -infections, defined as any infection following treatment for COVID - 19. Secondary endpoints included the duration of corticosteroid use, length of hospital stay, ICU length of stay, and mortality.

**Results:**

Of the 104 patients, 73% had co-infections or secondary infections. Pre-SOC patients were more likely to receive >10 days of corticosteroids (71% vs 30%). Co-infections were present in 12% of patients (13% in pre-SOC vs 11% in post-SOC), secondary infections occurred in 61% of patients (74% in pre-SOC vs 53% in post-SOC). The most common causative organism of co-infections and secondary infections were Staphylococcus aureus in the pre-SOC group and Escherichia coli and Pseudomonas aeruginosa in the post-SOC group. The mean hospital length of stay was 43 days pre-SOC vs 33 days post-SOC with a mean ICU length of stay of 33 vs 29 days, respectively. Mortality rate was similar between the two groups (55% vs 58%).

**Conclusion:**

Differences in epidemiology and microbiology was seen pre and post implementation of dexamethasone in June, 2020. Higher rates of co-infections were seen with this prolonged use of corticosteroids pre-SOC but it is unclear whether patients developed more co-infections as result of extended corticosteroid use, a longer hospital stay, or other factors. Further studies are needed to assess the optimal duration of corticosteroid use in this patient population with consideration to weigh benefit vs risk.

**Disclosures:**

**All Authors**: No reported disclosures

